# Identification of Hub Gene GRIN1 Correlated with Histological Grade and Prognosis of Glioma by Weighted Gene Coexpression Network Analysis

**DOI:** 10.1155/2021/4542995

**Published:** 2021-11-19

**Authors:** Aoran Yang, Xinhuan Wang, Yaofeng Hu, Chao Shang, Yang Hong

**Affiliations:** ^1^Department of Neurosurgery, Shengjing Hospital, China Medical University, Shenyang, Liaoning, China; ^2^Department of Neurobiology, School of Life Science, China Medical University, Shenyang, Liaoning, China

## Abstract

The function of glutamate ionotropic receptor NMDA type subunit 1 (GRIN1) in neurodegenerative diseases has been widely reported; however, its role in the occurrence of glioma remains less explored. We obtained clinical data and transcriptome data from the Gene Expression Omnibus (GEO) and The Cancer Genome Atlas (TCGA). Hub gene's expression differential analysis and survival analysis were conducted by browsing the Gene Expression Profiling Interactive Analysis (GEPIA) database, Human Protein Atlas database, and LOGpc database. We conducted a variation analysis of datasets obtained from GEO and TCGA and performed a weighted gene coexpression network analysis (WGCNA) using the R programming language (3.6.3). Kaplan-Meier (KM) analysis was used to calculate the prognostic value of GRIN1. Finally, we conducted Gene Ontology (GO) and Kyoto Encyclopedia of Genes and Genomes (KEGG) enrichment analyses. Using STRING, we constructed a protein–protein interaction (PPI) network. Cytoscape software, a prerequisite of visualizing core genes, was installed, and CytoHubba detected the 10 most tumor-related core genes. We identified 185 differentially expressed genes (DEGs). GO and KEGG enrichment analyses illustrated that the identified DEGs are imperative in different biological functions and ascertained the potential pathways in which the DEGs may be enriched. The overall survival calculated by KM analysis showed that patients with lower expression of GRIN1 had worse prognoses than patients with higher expression of GRIN1 (*p* = 0.004). The GEPIA and LOGpc databases were used to verify the expression difference of GRIN1 among GBM, LGG, and normal brain tissues. Ultimately, immunohistochemical assay results showed that GRIN1 was detected in normal tissue and not in the tumor specimens. Our results highlight a potential target for glioma treatment and will further our understanding of the molecular mechanisms underlying the treatment of glioma.

## 1. Introduction

Glioma—the most detrimental intracranial tumor—originates from the neuroepithelial tissue [1]. It occurs in approximately eight in 100,000 adults every year; young people are at a higher risk of certain phenotypes of glioma [2].

The World Health Organization (WHO) had categorized four pathological patterns of glioma. Among them, Category WHO VI has the highest malignant degree and incidence. The treatment strategy of glioma used to be excision radiotherapy and chemotherapy [3]. However, the potential treatments for glioma have inevitable side effects and limitations. For example, temozolomide (TMZ) chemotherapy is limited by the promoter methylation status of MGMT [4]. However, even when the most aggressive clinical methods are applied, the median survival time of glioma patients is still merely 12–15 months [5].

Therefore, numerous studies are being conducted on the molecular mechanism of glioma because of the unsatisfactory outcome of conventional treatments and the poor prognosis of glioma. Glutamate ionotropic receptor NMDA type subunit 1 (GRIN1) is an isoform of the glutamate receptor channel superfamily; it plays a crucial role in cooperating with NR2 (A-D) or NR3 (A-B) subunits of N-methyl-D-aspartate receptor and operates as a heteromultimer to form the ligand-gated ion channel and increase the plasticity of synapses [6]. *GRIN1* is located on chromosome band 9q34.3, a locus most associated with the occurrences of various diseases [7, 8]. In the past decade, numerous studies [9, 10] have shown that GRIN1 dysfunction causes various neurodegenerative diseases and mental illness. In addition, GRIN1 modulates the progression of some tumors [11]; however, no correlative research regarding its function and mechanism in the occurrence of glioma has been reported.

In this study, we collected data from The Cancer Genome Atlas (TCGA) [12] and Gene Expression Omnibus (GEO) to conduct variation analysis and weighted gene coexpression network analysis (WGCNA). We obtained 185 core genes by taking the intersection from the WGCNA and variation analysis results of TCGA and GEO and subsequently visualized the outcomes by plotting a Venn diagram. Using R programming language, we conducted the Gene Ontology (GO) [13] and Kyoto Encyclopedia of Genes and Genomes (KEGG) [14] enrichment analyses. Our results revealed the potential sites and pathways that GRIN1 acts upon and validated the diagnostic and prognostic values of GRIN1 in glioma. We believe that GRIN1 can be applied as a novel treatment target for the treatment of glioma.

## 2. Materials and Methods

### 2.1. Datum Origins

Microarray data were derived from the GEO database, which included a gene expression profile dataset (GSE109857) and a platform profile of GPL6480 Agilent-014850 Whole Human Genome Microarray 4x44K G4112F. The GSE109857 dataset contained 225 samples, 222 of which were glioma samples, whereas three were normal samples. We obtained transcriptome data of 174 glioma cases from the National Cancer Institute (NIH) GDC Data Portal. Data of 599 clinical samples were also abstracted from the NIH GDC Data portal.

### 2.2. Filtration and Annotation of Data

Annotation of the GEO datasets is necessary before conducting variation analysis and WGCNA when the data are enormous, obscure, and unmatched. The datasets downloaded from the GEO website were in two files: the platform files and the probe matrix file. The platform files and the probe matrix files were prepared as the input documents, and then, the PERL (version 5.32.1) arithmetic software was operated [15]. PERL processed the data by detecting the gene name and matching it with its specific probe according to the relationship between the gene name and the probe matrix, ultimately transforming the probe matrix into the gene matrix.

### 2.3. Detection of Differentially Expressed Genes (DEGs) and Glioma-Related Genes

EdgR (version 3.32.1) and limma [16] (version: 3.46.0) packages in Bioconductor were utilized to process the data and analyze DEG expression. We divided the genes into two groups with the different expression trends based on log fold change (FC) and calculated the mean value of the expressed genes in glioma to obtain the *t* value. We calculated the *p* value based on the *t* value and adjusted it using the false discovery rate (FDR) method. The DEGs were filtered under the condition of ∣logFC | >1 and adjusted *p* < 0.05. Moreover, we visualized DEGs by plotting heatmaps and volcano plots using the heatmap and ggplot packages in R, respectively.

To identify the glioma-related gene modules, we conducted WGCNA based on R programming language (3.6.3). The GO.db (version 3.12.1), preprocessCore (version 1.52.1), impute (version 1.64.0), and limma packages in Bioconductor were used to save and process the obtained datasets. The WGCNA package [17] was applied to identify the highly cooperative genes. Using the coefficient of association and the corresponding *p* value, we obtained several modules that reflected the relationships between tumor tissues and normal ones.

According to all modules we obtained from variation analysis and WGCNA, we selected the best glioma-related modules based on the most conspicuous coefficient of association and fetched information regarding the genes from the modules. The VennDiagram [18] package in R should be installed to identify the intersection among modules and plot the Venn diagram to visualize the consequences of the intersection.

### 2.4. Function Cognition and Pathway Enrichment Analysis

To detect how the glioma-related genes functioned in glioma and which sites and pathways they may act on, GO and KEGG enrichment analyses were conducted. Dose [19] (3.16.0), clusterProfiler [20] (3.18.1), and enrichplot (1.10.2) packages in Bioconductor and colorspace, stringi, and ggplot2 packages in R were applied for analyses.

Results of GO and KEGG enrichment analyses were output as two diagrams: a bar plot and a bubble diagram. Diagrams of GO enrichment analysis included three parts—a molecular function (MF) section, a cellular component (CC) section, and a biological process (BP) section—whereas the KEGG results revealed several pathways and target sites where the DEGs may be enriched. The results of GO enrichment and KEGG enrichment analyses were calculated based on *p* < 0.05.

### 2.5. Configuration of the PPI Network and Developing a Network of Hub Genes

After filtering glioma-related genes, we attempted to identify the potential interaction among these genes and subsequently developed a protein–protein interaction (PPI) network. The hub genes were identified using STRING [21] (version 11.0) and the CytoHubba [22] plug-in in Cytoscape [23] software.

The application of Cytoscape software is aimed at abstracting DEGs encoding proteins and establishing a network scaffold. The CytoHubba plug-in can detect and locate 10 of the most relevant DEGs using the maximal clique centrality (MCC) and mark them in red (high correlation), orange (medium correlation), and yellow (low correlation) colors based on their correlation with glioma.

### 2.6. Correlation Analysis between the Target Gene and Glioma

GRIN1 was selected from the 10 hub genes and selected as the optimum gene. Confirmation regarding whether it is associated with the occurrence of gliomas is required. Module GEPIA2 [24] in the Gene Expression Profiling Interactive Analysis (GEPIA) database provides a macroperspective of the difference in the gene expression between glioma and normal tissues.

### 2.7. Clinical Correlation Analysis

In this study, to identify the difference in the expression of GRIN1 between different sexes and ages of glioma patients, we conducted two different clinical correlation analysis including ages and sexes. The limma package in Bioconductor was applied to perform a comprehensive analysis between two different groups, whereas the ggpubr package in R was installed and utilized to visualize the comparison. The results of the comparison analysis were calculated based on the *p* value.

### 2.8. Verification of the Protein Expression of Hub Genes in Tissue Based on the Human Protein Atlas (HPA) Database, GEPIA Website, and LOGpc Database

The expression of core genes was verified by browsing the Human Protein Atlas (HPA) database to identify the differential expression of GRIN1 between the normal tissue and glioma tissue. HPA contains abundant data; more than 26,000 antibodies have been collected, and all results are immunohistochemical results. The HPA database is free for use for researchers. We input the hub gene into the website and chose the tissue module and pathology module to run the analysis. The cerebral cortex was selected, and the result with immunohistochemical images was generated automatically. The antibody serial number chosen was HPA 067773 in both the normal tissue and tumor tissue.

The survival and survminer packages in R were applied to analyze the overall survival (OS) using the best cut-off criteria based on the gene samples. We divided the samples based on the expression level of the target genes and conducted the KM analysis to check the difference in the survival rate between the groups. A *p* value of <0.05 was considered statistically significant. Subsequently, a survival curve diagram was plotted to visualize the survival analysis results. The GEPIA [25] database was used to analyze the expression difference among GBM, LGG, and normal brain tissues. In order to understand the correlation between GRIN1 and glioma more comprehensively, we consulted the LOGpc [26] database to verify the expression difference of GRIN1 in a high-grade glioma and a low-grade glioma.

### 2.9. Tissue Sample Collection

Human specimens of glioma and normal brain tissues were collected from China medical University Shengjing Hospital (Shenyang, China). All patients were informed of the use of tissues. The study protocol was approved by ethics committee of Shengjing Hospital. Tissues are preserved well in liquid nitrogen after surgery and divided into two groups according to the 2007 WHO classification guidelines of tumors in the central nervous system: WHO I-II (*n* = 3) and WHO III-IV (*n* = 3). Normal human brain tissue (*n* = 1) was obtained from a cerebrovascular malformation case, which was considered the negative control.

### 2.10. Immunohistochemical (IHC)

Frozen tissues were paraffin-embedded and then sliced to mount on slides. Slides were preprocessed by dewaxing and antigen repairing; a primary antibody against protein was used (GRIN1, 1 : 100 dilutions, Abclonal, China). Slides were preserved overnight at 4°C. Subsequently, we incubated slides with a secondary antibody and streptavidin peroxidase (MXB biotechnologies, China). Development using DAB and nuclear counterstaining by hematoxylin was done in the final step.

## 3. Results

### 3.1. Variation Analysis and WGCNA of DEGs

**Figure 1:**
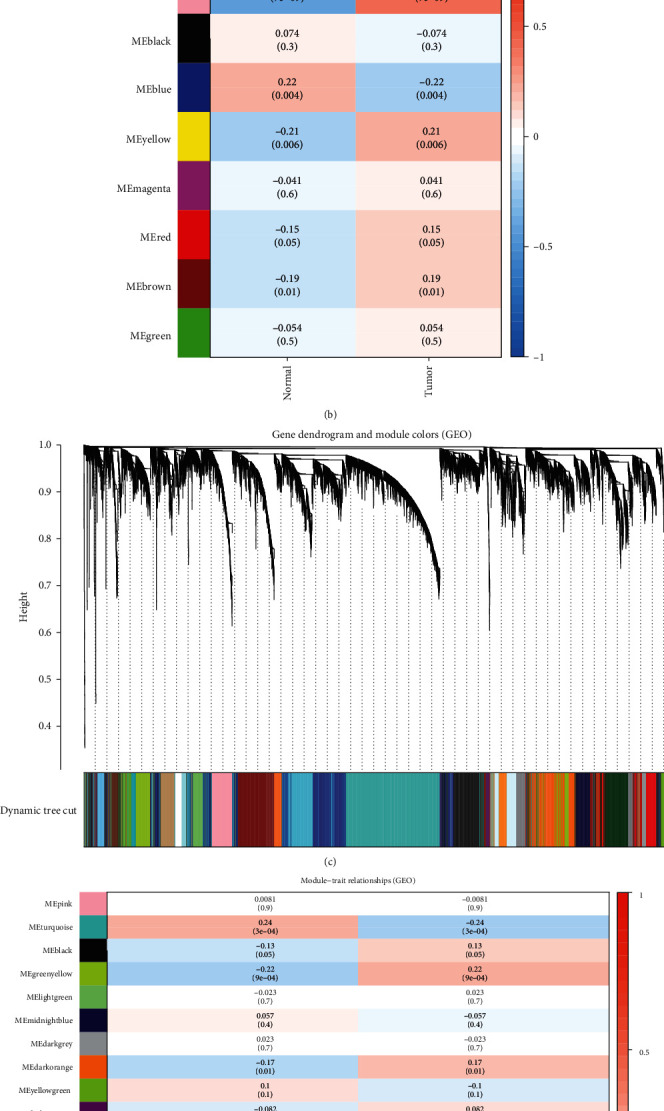
DEGs are separated into different modules based on the *p* value and the relationships between DEGs and the clinical information in TCGA-GLIOMA and GEO-GLIOMA are shown. (a) A cluster dendrogram was constructed, and the relationship among the coexpression genes is shown in a hierarchy cluster based on the 1-TOM matrix. Each color represents a module. (b) Module-trait relationships (TCGA). The lengthways grids represent a module with different colors. The crosswise grids represent the clinical relevance (normal and tumor). (c) A cluster dendrogram was constructed, and the relationship among the coexpression genes in a hierarchy cluster is shown based on the 1-TOM matrix. Each color represents a module. (d) Module-trait relationships (GEO). The lengthways grids represent a module in different colors. The crosswise grids represent the clinical relevance (normal and tumor).

To identify DEGs in the TCGA datasets and GEO datasets, variation analysis and WGCNA must be conducted, and then, a network based on the gene's cooperation relationship must be constructed. To visualize the results, we placed the gene clusters into different modules by plotting dendrograms (Figures 1(a) and 1(c)); meanwhile, module-trait diagrams were also obtained based on R. These dendrograms illustrated the association of the gene cluster between the normal and tumor tissues. After WGCNA of TCGA datasets, a diagram containing 9 colors representing different modules were constructed (Figure 1(b)), whereas for the GEO datasets, the results included 27 colors representing different modules (Figure 1(d)). The turquoise module was declared to have the highest association with the normal tissue (*r* = 0.73, *p* = 9*e* − 31) and tumor tissue (*r* = −0.73, *p* = 9*e* − 31) after TCGA analysis. A common phenomenon was observed in the GEO WGCNA results, wherein the turquoise module represents the most relevant relationship with the normal tissue (*r* = 0.24, *p* = 3*e* − 04) and tumor tissue (*r* = −0.24, *p* = 3*e* − 04).

**Figure 2:**
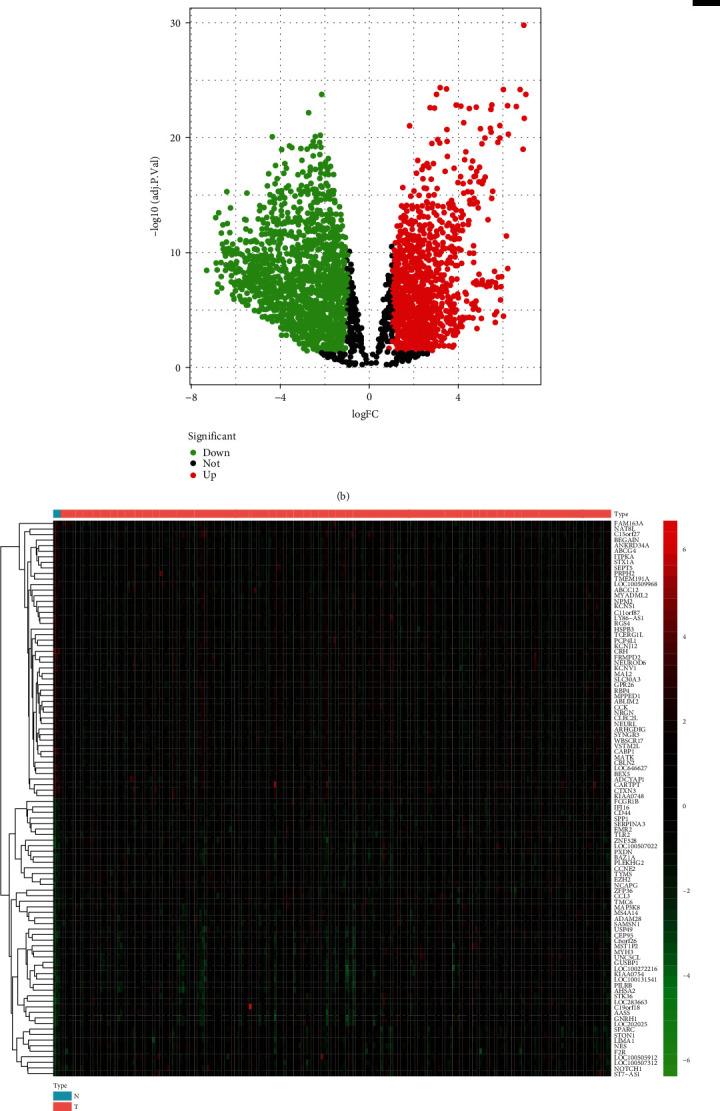
Identification of the expression of DEGs in the normal and tumor tissues in the TCGA dataset and GEO dataset. The criteria are as follows: ∣logFC | >1, adjusted *p* < 0.05. (a) Heatmap used to visualize the consequence of DEGs of TCGA. The red section represents the upregulation of DEGs in tumor, whereas the green section represents the upregulation of DEGs in normal tissue. (b) Volcano diagram based on the TCGA database. (c) Heatmap used to visualize the consequence of DEGs of GSE109857. The red section represents the upregulation of DEGs in tumor, whereas the green section represents the upregulation of DEGs in normal tissue. (d) Volcano diagram of the GSE109857 dataset.

The output of the TCGA and GEO analyses was heatmaps (Figures 2(a) and 2(c)) and volcano diagrams (Figures 2(b) and 2(d)). These diagrams exhibited solid evidence illustrating the relationships between DEGs and tissues. Two groups were divided based on the expression level in different tissues in both the heatmap and volcano plots.

### 3.2. Abstracting the Intersection of the DEGs

**Figure 3:**
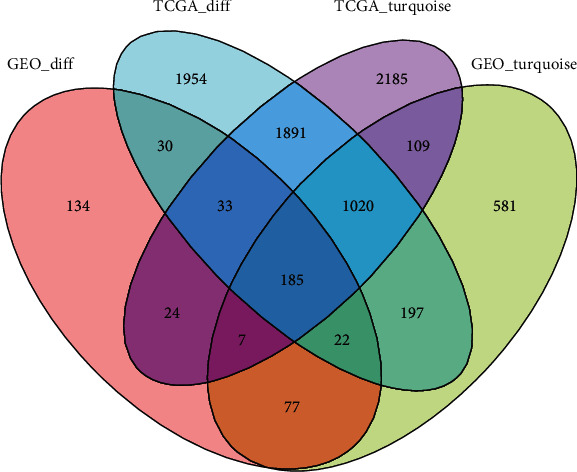
Venn diagram shows the intersection results of the variation analysis and WGCNA. A total of 185 DEGs were obtained and visualized.

Two module-trait diagrams and a pair of heatmaps and volcano diagrams were constructed using the results of variation analysis and WGCNA. To understand the intersection of the variation analysis and WGCNA, we imported packages of VennDiagram in R and plotted the Venn diagram. As shown in the Venn diagram (Figure 3), the intersection was located over 4 parts (the GEO_diff, TCGA_diff, TCGA turquoise, and GEO turquoise). A total of 185 genes were marked in this intersection.

### 3.3. Enrichment Analysis of DEGs

To gain a further understanding of how DEGs mobilized and worked in glioma, we conducted an enrichment analysis. Two types of enrichment analysis were performed based on R. Dose and clusterProfiler packages in Bioconductor were installed. The enrichment analysis provided a pair of bar plots and bubble diagrams.

**Figure 4:**
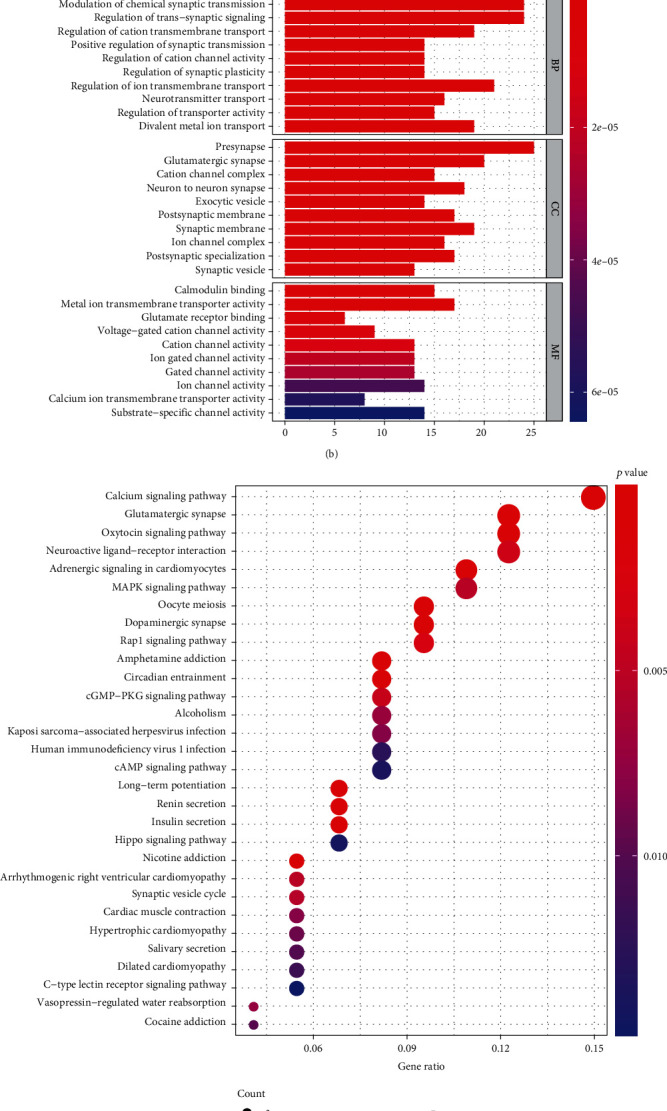
GO enrichment analysis of the turquoise module and the KEGG enrichment analysis of the turquoise module. Results were visualized using a bar plot and bubble diagram. (a) Bar plot. Gradients of colors are based on the adjusted *p* value. (b) Bubble plot. Gradients of colors are based on the adjusted *p* value. The size of the circle represents the gene number. (c) Bar plot. Gradients of colors are based on the adjusted *p* value. (d) Bubble plot. Gradients of colors are based on the adjusted *p* value. The size of the circle represents the gene number.

A bubble diagram (Figure 4(a)) and bar plot (Figure 4(b)) were constructed based on the results of the GO enrichment analysis which included three parts—molecular function (MF), CC, and BP. DEGs play a significant role in modulating the chemical synaptic transmission and regulating the *trans*-synaptic signaling in the BP section. In the CC section, we observed that DEGs were enriched on presynapse and in the MF section, and DEGs are involved in the process of a metal ion transmembrane transporter. The KEGG enrichment analysis results were used to construct a bubble diagram (Figure 4(c)) and a bar plot (Figure 4(d)), which both illustrated that DEGs are enriched in the calcium signaling pathway.

### 3.4. Configuration of the PPI Network and Localization of Hub Genes

**Figure 5:**
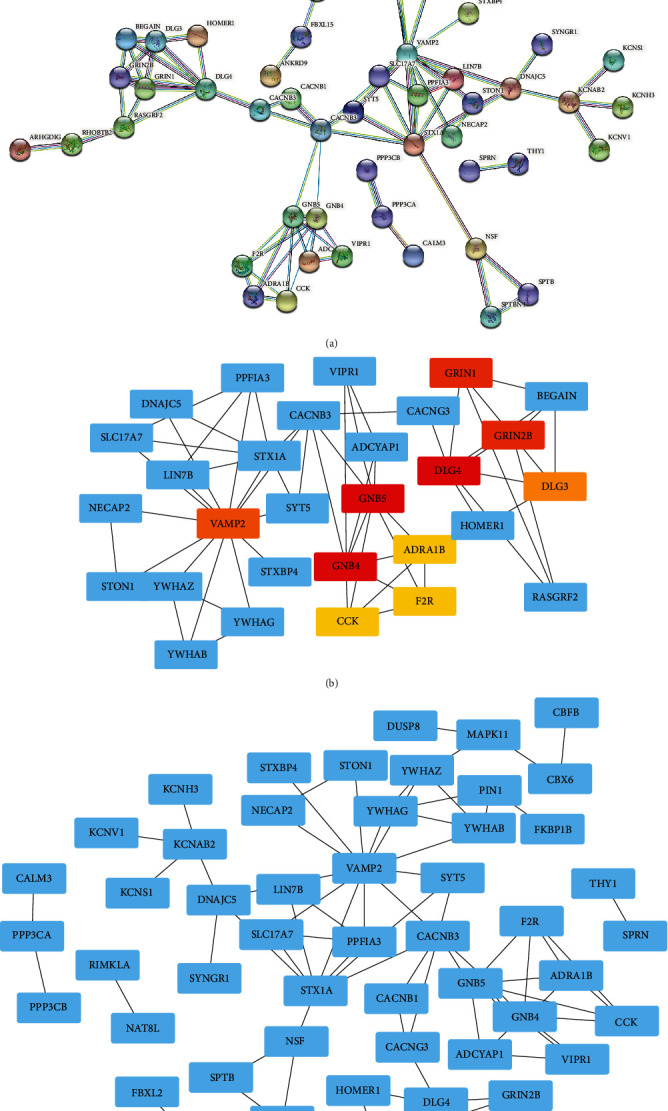
The establishment and visualization of the PPI network and identification of hub genes. (a) A PPI network was constructed by the STRING website; each circle represents a single gene. Genes are linked by line segments that highlight the association among them. (b) Hub genes were identified by CytoHubba based on the MCC algorithm. Red color represents a high level of hub genes, whereas orange color represents medium level and yellow low level of hub genes. (c) Blue nodes represent gene names. Relationships among genes are illustrated by the line connection.

A PPI network can only be established once the connections among the DEGs are clarified. The results obtained from STRING are shown in Figure 5(a). Cytoscape software was used to clarify the PPI network. CytoHubba, a plug-in in Cytoscape, selects and marks 10 of the most relevant DEGs with colors based on the MCC, which includes YWHAG, GRIN1, GRIN2B, CACNB3, STX1A, DLG4, VAMP2, BEGAIN, ADRA1B, and RASGRF2. Results of visualization of the PPI network are shown in Figures 5(b) and 5(c).

### 3.5. Survival Analysis of GRIN1 and Comparison of GRIN1 in Different Ages and Genders

**Figure 6:**
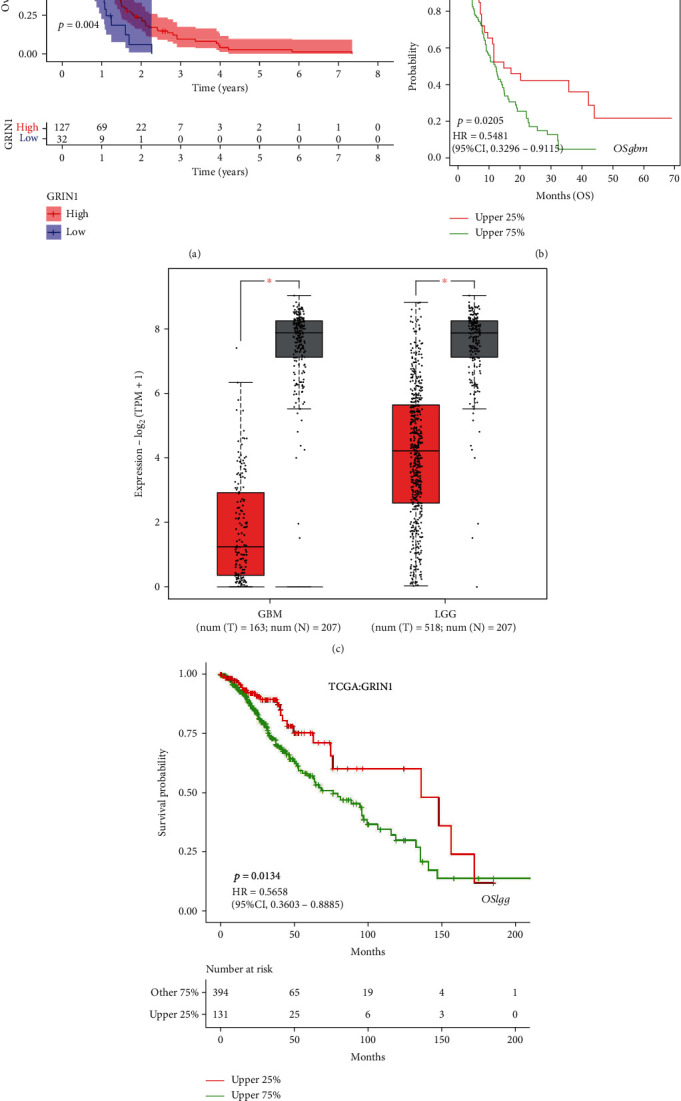
Expression difference of GRIN1 in glioma and normal tissues based on the GEPIA database and LOGpc database. Survival status of differential expression of GRIN1. (a) OS based on GRIN1 expression levels. Calculated using the *p* value based on Kaplan-Meier (KM) analysis. (b) OS based on GRIN1 expression levels in the GBM group by searching the LOGpc database. (c) Difference in GRIN1 expression among GBM, LGG, and normal tissue. Red represents GRIN1 expression in glioma, whereas gray represents GRIN1 expression in normal tissue. (d) OS based on GRIN1 expression levels in the LGG group by searching the LOGpc database.

The target gene GRIN1 was chosen to perform survival analysis. The survival and survminer packages were used for analysis. The curve graph (Figure 6(a)) that we obtained showed that patients with low expression of GRIN1 had worse prognosis than patients with higher expression (*p* = 0.004). Subsequently, we browsed the GEPIA database and verified the expression difference of GRIN1 between the high-grade glioma and low-grade glioma. Another box plot (Figure 6(c)) proved that the expression of GRIN1 differed among the high-grade glioma group, low-grade glioma group, and normal tissue, whereas the lower expression of GRIN1 represents higher grades of malignancy. Consequence by browsing, the LOGpc database also showed this difference. The curve graph in Figure 6(b) shows that in the high grade-glioma (WHO III-IV) group, patients with high expression of GRIN1 earned a better prognosis than patients with lower expression of GRIN1 (*p* = 0.0205). Another curve graph (Figure 6(d)) illustrated that in low-grade glioma (WHO I-II), patients with high expression of GRIN1 also earned a better prognosis than patients with lower expression of GRIN1 (*p* = 0.0134). Thus, the lower the expression of GRIN1, the worse the prognosis of patients.

**Figure 7:**
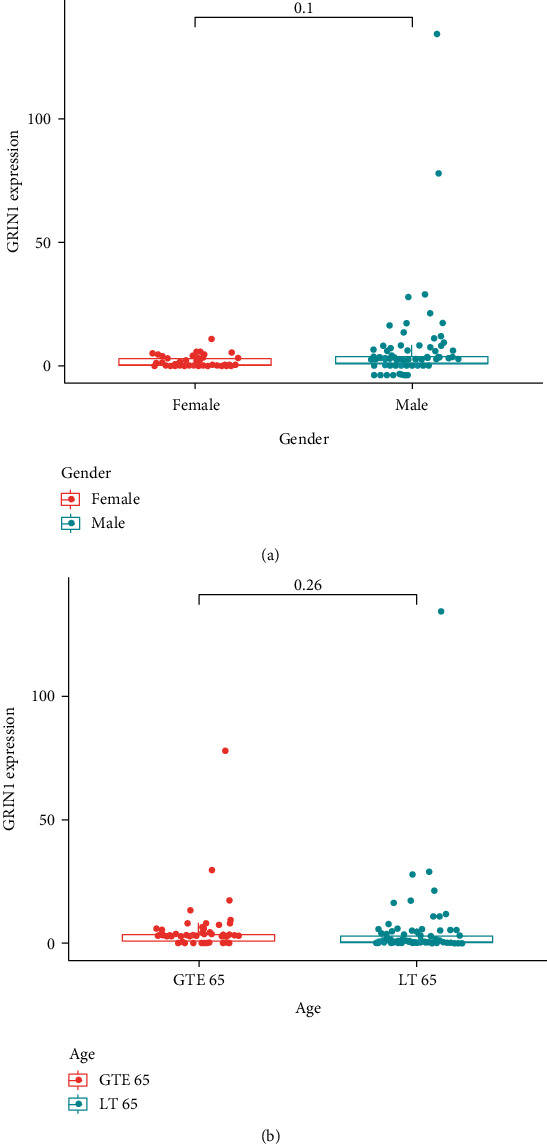
Verification of the expression of GRIN1 in different sexes and ages. (a) Comparison of the expression between females and males based on the *p* value. (b) Comparison of the expression between groups of patients greater than 65 ages and less than 65 ages.

To analyze whether GRIN1 is expressed differently in different ages and sexes, we separated the samples into two groups: the age comparison group (two more groups were separated by the boundary of 65 ages) and the sex comparison group. The limma and ggpubr packages of R were used for analysis. There was no difference in the GRIN1 expression between males and females in the glioma tissue (Figure 7(a), *p* = 0.1). Furthermore, no difference was observed between people younger than 65 and people older than 65 (Figure 7(b), *p* = 0.26).

### 3.6. Expression of the Hub Genes in Protein and Glioma Tissue

**Figure 8:**
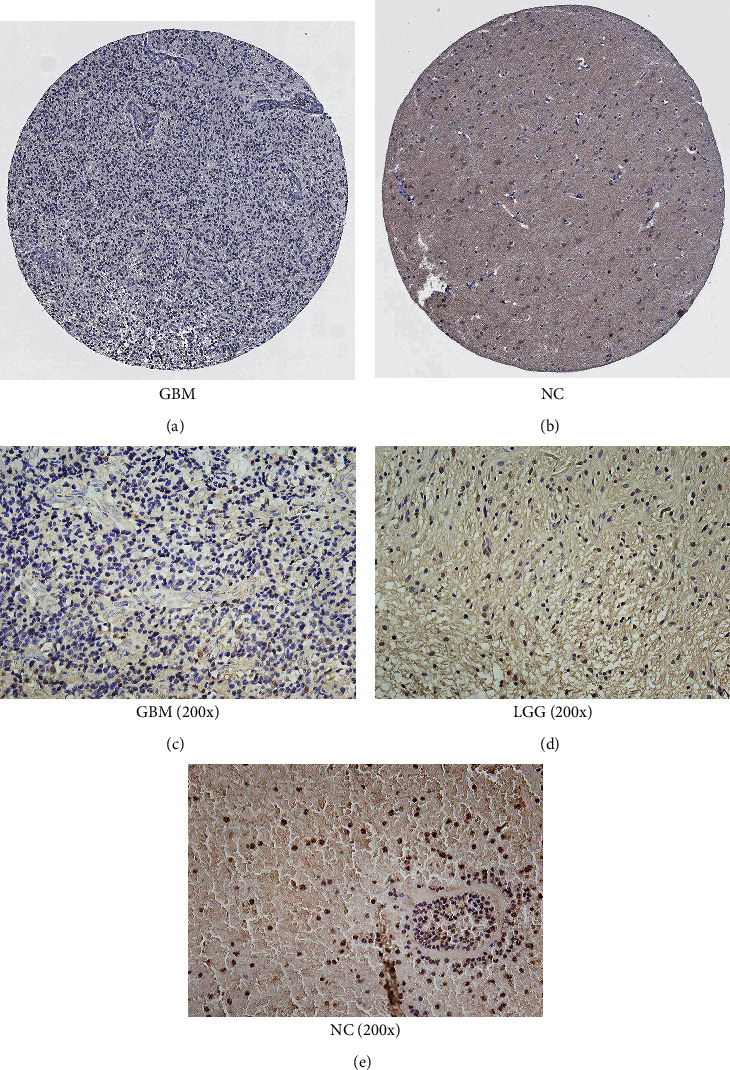
IHC results of GRIN1 protein levels obtained by searching the HPA database and conducting IHC experiment. (a) Protein levels of GRIN1 in the glioma tissue of the human brain (antibody HPA067773, staining: not detected; intensity: negative; quantity: none). (b) Protein levels of GRIN1 in the normal tissue of the human brain (antibody HPA067773, staining: low; intensity: weak; quantity: >75%-25%). (c–e) Images (200x) of hematoxylin-stained section with immunohistochemical detection of GRIN1 in GBM tissues, LGG tissues, and normal brain tissues.

By searching the HPA database, we inputted *GRIN1* as the target gene into the website. The output showed a significant difference, wherein GRIN1 was not detected in the GBM tissue (Figure 8(a)) and detected in the normal brain tissue. (Figure 8(b)). To confirm the expression difference of GRIN1 in glioma tissues and normal brain tissues, we conducted IHC. Results indicated that GRIN1 was in a low expression status in the GBM tissue (Figure 8(c)) and LGG tissue (Figure 8(d)) compared to the normal brain tissue (Figure 8(e)).

## 4. Discussion

Glioma is a type of refractory intracranial tumor, which can occur at any location in the central nervous system such as the brainstem, ventricle, cerebellum, corpus callosum, and basal ganglia. Numerous molecular studies have been conducted because of the tremendous harm that it causes and the poor curative effect of conventional neurosurgery and chemotherapy. A previous study [27] confirmed that with the mediation of the upstream miRNA or ceRNA, downstream genes can exhibit a synergistic effect, taking part in glioma growth, proliferation, and invasion by changing the expression level. Because of the differences in gene characteristics, the high or low expression of genes can both be involved in the process of glioma, thereby accelerating glioma progression [28, 29].

In this study, the GO enrichment analysis outcomes showed that the 185 DEGs were mostly related to the modulation of chemical synaptic transmission and the regulation of the *trans*-synaptic signaling pathway within the BP section, and hub genes were enriched in presynapse in the CC section; in addition, they are involved in the process of the metal ion transmembrane transporter. The results of the KEGG enrichment analysis showed that the hub genes were enriched in the calcium signaling pathway. By establishing a protein–protein interaction (PPI) network and using Cytoscape software, we identified GRIN1 as a potential biomarker for glioma. Subsequently, we conducted immunohistochemical assay and browsed the Human Protein Atlas (HPA) to compare the results and identify differences in protein expression between the normal and tumor tissues. Based on these results, we showed that the dysregulation of GRIN1 could lead to the occurrence of glioma, and its low expression is strongly associated with poor prognosis of patients with glioma.

GRIN1 is located on chromosome band 9q34.3. In diseases of the nervous system, GRIN1 has frequently been reported in neurodegenerative diseases, while it has been reported to a lower degree in glioma. Thus, the specific mechanism of GRIN1 expression in the growth, proliferation, and invasion of glioma is still unclear. Thus, we need more experiments to uncover this mystery.

Our study has some inevitable limitations. First, data obtained from the TCGA database and GEO database are not complete and the samples are insufficient, which may lead to a low statistical power. Moreover, the clinical stages of the samples we obtained from the GEO database were WHO III; thus, in this research, we only compared the differences between ages and sexes without analyzing the differences among different glioma grades and prognosis. Second, this study is merely a bioinformatics research based on data screening and analysis. Although we have estimated the molecular mechanism and the prognostic value of GRIN1, further systematic studies on the mechanism of GRIN1 at the molecular, cellular, and biological levels based on experimental techniques such as western blotting need to be performed.

## Data Availability

Transcriptome data and clinical data can be downloaded from https://portal.gdc.cancer.gov/. The gene expression profile dataset (GSE109857) can be downloaded from https://www.ncbi.nlm.nih.gov/geo/query/acc.cgi?acc=GSE109857. The platform profile of GPL6480 Agilent-014850 Whole Human Genome Microarray 4x44K G4112F can be downloaded from: https://www.ncbi.nlm.nih.gov/geo/query/acc.cgi?acc=GPL6480

## References

[1] Jiang Y., Uhrbom L. (2012). On the origin of glioma. *Upsala Journal of Medical Sciences*.

[2] Ostrom Q. T., Kinnersley B., Armstrong G. (2018). Age-specific genome-wide association study in glioblastoma identifies increased proportion of 'lower grade glioma'-like features associated with younger age. *International Journal of Cancer*.

[3] Jiang T., Nam D.-H., Ram Z. (2021). Clinical practice guidelines for the management of adult diffuse gliomas. *Cancer Letters*.

[4] Whitelaw B. C. (2019). How and when to use temozolomide to treat aggressive pituitary tumours. *Endocrine-Related Cancer*.

[5] Mountz J., Ahmed R., Oborski M., Lieberman F., Hwang M. (2014). Malignant gliomas: current perspectives in diagnosis, treatment, and early response assessment using advanced quantitative imaging methods. *Cancer Management and Research*.

[6] Monyer H., Sprengel R., Schoepfer R. (1992). Heteromeric NMDA receptors: molecular and functional distinction of subtypes. *Science (New York, N.Y.)*.

[7] Okur V., Nees S., Chung W. K., Krishnan U. (2018). Pulmonary hypertension in patients with 9q34.3 microdeletion-associated Kleefstra syndrome. *American journal of medical genetics: Part A*.

[8] Bonati M. T., Castronovo C., Sironi A. (2019). 9q34.3 microduplications lead to neurodevelopmental disorders through EHMT1 overexpression. *Neurogenetics*.

[9] Intson K., van Eede M. C., Islam R. (2019). Progressive neuroanatomical changes caused by _Grin1_ loss-of-function mutation. *Neurobiology of Disease*.

[10] Scala M., Amadori E., Fusco L. (2019). Abnormal circadian rhythm in patients with _GRIN1_ -related developmental epileptic encephalopathy. *European journal of paediatric neurology: EJPN : official journal of the European Paediatric Neurology Society*.

[11] Zhuang M. Q. (2020). G protein regulated inducer of neurite outgrowth 1 is a potential marker for lung cancer prognosis. *Journal of Biological Regulators and Homeostatic Agents*.

[12] Wang Z., Jensen M. A., Zenklusen J. C. (2016). A practical guide to The Cancer Genome Atlas (TCGA). *Methods in molecular biology (Clifton, N.J.)*.

[13] Ashburner M., Ball C. A., Blake J. A. (2000). Gene Ontology: tool for the unification of biology. *Nature Genetics*.

[14] Kanehisa M., Goto S. (2000). KEGG: Kyoto Encyclopedia of Genes and Genomes. *Nucleic Acids Research*.

[15] Fourment M., Gillings M. R. (2008). A comparison of common programming languages used in bioinformatics. *BMC Bioinformatics*.

[16] Gentleman R. (2005). *Bioinformatics and Computational Biology Solutions Using R and Bioconductor*.

[17] Langfelder P., Horvath S. (2008). WGCNA: an R package for weighted correlation network analysis. *BMC Bioinformatics*.

[18] Chen H., Boutros P. C. (2011). VennDiagram: a package for the generation of highly-customizable Venn and Euler diagrams in R. *BMC Bioinformatics*.

[19] Ritz C., Baty F., Streibig J. C., Gerhard D. (2015). Dose-response analysis using R. *PLoS One*.

[20] Yu G., Wang L.-G., Han Y., He Q.-Y. (2012). clusterProfiler: an R package for comparing biological themes among gene clusters. *Omics: a journal of integrative biology*.

[21] Szklarczyk D., Gable A. L., Nastou K. C. (2021). The STRING database in 2021: customizable protein-protein networks, and functional characterization of user-uploaded gene/measurement sets. *Nucleic Acids Research*.

[22] Chin C.-H. (2014). cytoHubba: identifying hub objects and sub-networks from complex interactome. *BMC systems biology*.

[23] Chin C.-H., Chen S.-H., Wu H.-H., Ho C.-W., Ko M.-T., Lin C.-Y. (2019). Cytoscape StringApp: network analysis and visualization of proteomics data. *Journal of Proteome Research*.

[24] Tang Z., Kang B., Li C., Chen T., Zhang Z. (2019). GEPIA2: an enhanced web server for large-scale expression profiling and interactive analysis. *Nucleic Acids Research*.

[25] Tang Z., Li C., Kang B., Gao G., Li C., Zhang Z. (2017). GEPIA: a web server for cancer and normal gene expression profiling and interactive analyses. *Nucleic acids research*.

[26] Dong H., Wang Q., Li N. (2020). OSgbm: an online consensus survival analysis web server for glioblastoma. *Frontiers in Genetics*.

[27] Zhou Q., Liu J., Quan J., Liu W., Tan H., Li W. (2018). MicroRNAs as potential biomarkers for the diagnosis of glioma: a systematic review and meta-analysis. *Cancer Science*.

[28] Wu D.-M., Hong X.-W., Wen X. (2019). MCL1 gene silencing promotes senescence and apoptosis of glioma cells via inhibition of the PI3K/Akt signaling pathway. *IUBMB Life*.

[29] Zhang F., Ruan X., Ma J. (2020). DGCR8/ZFAT-AS1 promotes CDX2 transcription in a PRC2 complex-dependent manner to facilitate the malignant biological behavior of glioma cells. *Molecular therapy: the journal of the American Society of Gene Therapy*.

